# Bruxism Simulation in Aligner Therapy: Effects on Restored Posterior Teeth

**DOI:** 10.3390/jcm14217877

**Published:** 2025-11-06

**Authors:** Amelia Anita Boitor (Andreica), Adriana Objelean, Cristina Gasparik, Alexandru Victor Burde, Horațiu Alexandru Colosi, Diana Dudea

**Affiliations:** 1 Department of Prosthetic Dentistry and Dental Materials, Iuliu Hatieganu University of Medicine and Pharmacy (UMFIH), 400006 Cluj-Napoca, Romania; amelia.boitor@elearn.umfcluj.ro (A.A.B.); gasparik.cristina@umfcluj.ro (C.G.); ddudea@umfcluj.ro (D.D.); 2 Department of Dental Technology, Faculty of Nursing and Life Sciences, Iuliu Hatieganu University of Medicine and Pharmacy (UMFIH), 400012 Cluj-Napoca, Romania; burde.alexandru@umfcluj.ro; 3 Department of Medical Informatics and Biostatistics, Iuliu Hatieganu University of Medicine and Pharmacy (UMFIH), 400012 Cluj-Napoca, Romania

**Keywords:** bruxism, aligners, composite restorations, chewing simulation, digital orthodontics, treatment planning, orthodontics, dentistry

## Abstract

**Background/Objectives:** Parafunctional habits such as bruxism generate high occlusal forces that can significantly compromise the performance of dental restorations during orthodontic treatment. This ex vivo study aimed to evaluate the surface wear of Class II composite restorations and the integrity of clear aligners (CAs) under simulated parafunctional loading. **Methods:** Thirty-four posterior teeth restored with composite materials were subjected to either normal masticatory forces or high-intensity cyclic forces mimicking bruxism while being fitted with orthodontic aligners. The collected experimental data were analyzed using *R* (version 4.3) under the Jamovi project (version 2.5.3). Differences between groups were assessed using paired samples *t*-tests and Wilcoxon tests for paired samples, with robust *t*-tests applied when data normality could not be confirmed. Statistical significance was set at α = 0.05. **Results:** Parafunctional loading led to significantly greater surface degradation of restorations and increased aligner wear. Compared with functional forces, RMS errors were substantially higher under parafunctional forces (33.5 vs. 21.5 units; *p* < 0.001), indicating reduced positional accuracy. Aligner thickness decreased more under parafunctional conditions (0.0304 mm) than under normal function (0.0122 mm), with all comparisons showing high statistical significance and large effect sizes. **Conclusions:** Parafunctional forces were found to significantly increase surface wear in Class 2 resin composite restorations during clear aligner therapy. Simulated bruxism also compromised aligner integrity, indicating the need for more durable materials and tailored treatment strategies for patients with bruxing habits. These findings highlight the importance of selecting durable restorative and aligner materials for bruxer patients to ensure long-term treatment success.

## 1. Introduction

Orthodontic aligners have revolutionized modern dentistry, providing an aesthetically pleasing and comfortable alternative to conventional braces. These custom-fabricated aligners, typically made from thermoplastic polymers such as polyethylene terephthalate glycol-modified (PET-G) or polyurethane, are designed to move teeth into their desired positions while ensuring patient compliance and satisfaction [1].

Bruxism, a condition characterized by involuntary grinding or clenching of teeth, is a significant factor contributing to the wear and tear of both natural teeth and restorative materials [2]. The repetitive forces exerted during bruxism episodes can lead to microfractures, material degradation, and even the failure of dental restorations over time [3].

Aligners, when used by bruxer patients, introduce an additional variable into this dynamic. On the one hand, aligners may serve as a protective barrier. Clear aligners (CAs) have been shown to reduce sleep bruxism activity and muscular hyperactivity, likely due to their occlusal coverage, neuromuscular modulation, and their ability to distribute occlusal forces more evenly. Thus, they may shield restorations from direct impact [4,5]. However, the thermoplastic materials of aligners, although durable, are not immune to deformation or wear under excessive forces associated with high-impact parafunctional activities. This raises questions about their effectiveness in such scenarios [4]. Resin composite fillings are commonly used for their aesthetic appeal and ability to bond with tooth structures. These fillings are particularly susceptible to these forces. The longevity of these fillings in patients with parafunctional masticatory forces is, therefore, a legitimate concern in dental practice.

Resin-based composites are frequently used to restore interproximal surfaces of posterior teeth due to their aesthetic appeal and conservative application. However, their mechanical durability—particularly resistance to wear and fracture—may be compromised in high-load conditions such as those caused by bruxism, where repetitive stress accelerates material fatigue and increases the risk of failure [6,7]. Evaluating how aligners influence the longevity of these fillings in patients with bruxism is clinically relevant. This evaluation is also crucial for optimizing treatment protocols and patient outcomes [2,8,9].

The impact of aligners on dental restorations, especially Class II composite fillings, remains underexplored. This is particularly true in patients with parafunctional masticatory forces. Therefore, this study aimed to evaluate the effect of simulated functional and parafunctional forces on Class II restored teeth undergoing orthodontic treatment with aligners. By simulating masticatory forces ex vivo, we sought to provide insights into the mechanical performance and wear patterns of these restorations. The conditions represent both normal forces and those related to bruxism. Understanding these interactions is essential for optimizing treatment strategies. It also helps improve long-term outcomes for patients with restorative and orthodontic needs. The null hypotheses of this study were:

(1) Simulated parafunctional forces have a similar effect as functional forces on the integrity of Class II restored teeth covered by orthodontic aligners.

(2) Simulated parafunctional forces have a similar effect as functional forces on the integrity of orthodontic aligners.

## 2. Materials and Methods

### 2.1. Experimental Design

For this study, the sample size was determined from a prior pilot study. This study helped us estimate a rather large effect size of 1 for the main hypothesis. The sample size was calculated using G*Power software version 3.1.9.7 (Kiel University Software, Germany). We planned to use a *t*-test family for matched pairs in our future analyses. With a desired statistical power (1 − β) of 0.8 and a significance level of 0.05, we calculated a minimally required sample size of 17 samples per group. The Root Mean Square (RMS) error was the primary endpoint guiding this calculation, representing the quantitative assessment of material loss and deformation in Class II restored teeth subjected to simulated functional and parafunctional forces. In total, 17 standardized Class II cavities were prepared, with one cavity per tooth.

Therefore, 34 extracted human molars were randomly divided into two groups:

Group 1 (Control), n = 17—Subjected to normal masticatory forces.

Group 2 (Bruxism Simulation): n = 17—Subjected to simulated parafunctional masticatory forces/bruxism forces.

**Figure 1:**
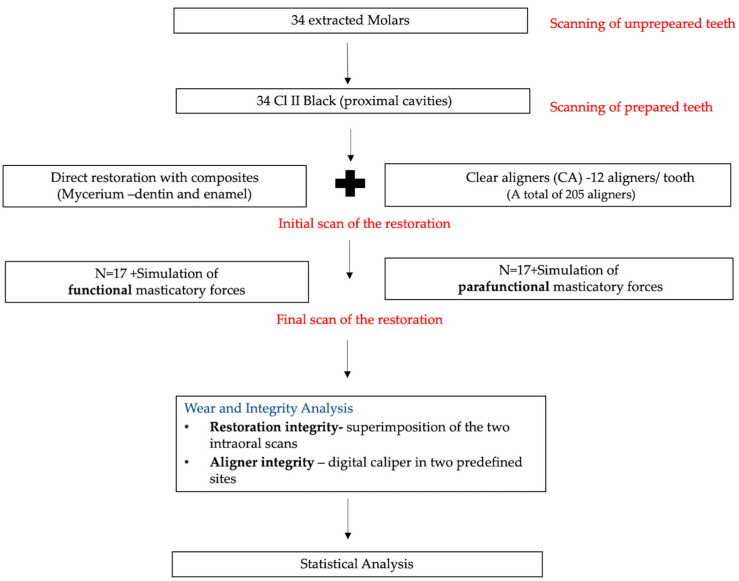
The workflow of the protocol used in the study.

The workflow of the protocol used is explained in the figure below (Figure 1).

### 2.2. Specimen Preparation and Restorative Procedure

The study protocol has been approved by the Research Ethics Committee of the Iuliu Hatieganu University of Medicine and Pharmacy, Cluj-Napoca, Romania (247/30 June 2021).

Thirty-four caries-free, fracture-free, unrestored human third molars were selected. Prior to experiments, all teeth were kept in 1% Chloramine T at 4 °C, then rinsed thoroughly and stored in distilled water until use. Each molar was scanned with an intraoral scanner (Medit i700, Medit Corp., Seoul, Republic of Korea) before and after cavity preparation to ensure cavity accuracy and standardization.

**Figure 2:**
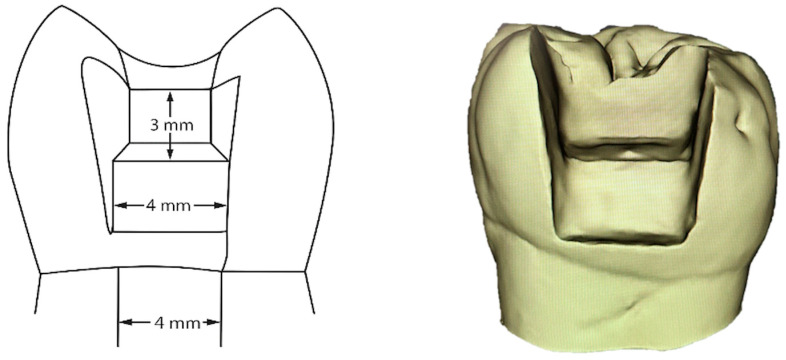
Graphical scheme and 3D scan of a sample tooth with Class II standardized cavity.

For standardized Class II cavities (mesio-occlusal or disto-occlusal), the teeth were placed in polyvinylsiloxane silicone material. This helped standardize their position during the preparation phase. An experienced operator prepared all cavities using a high-speed handpiece with water cooling and a 0.12 cylindrical bur (8882, Komet, Lemgo, Germany). After preparation, all cavity margins were finished with an Arkansas bur (649, Komet, Lemgo, Germany). The cavity dimensions were measured and standardized using Exocad GmbH software version 2.4 Plovdiv (Darmstadt, Germany). This ensured cavities were consistently sized at 3 mm occlusal depth, 4 mm buccal-lingual width, 2 mm proximal depth, and 4 mm width at the cervical rim. All proximal cavities had the gingival margin placed 1 mm above the cementoenamel junction (CEJ) (see Figure 2).

The prepared cavities were restored using the Stamp technique to ensure proper anatomical reproduction of the occlusal surface [10]. For the adhesive system, ENA Etch & Rinse One Bottle (Mycerium Dental, Lançon-Provence, France) [11] was used according to the manufacturer’s instructions.

The first composite layer was heated HRI Bio Function Dentine (Mycerium Dental, France) [12], applied as a 1 mm layer, followed by two 1.5 mm layers of heated HRI Bio Function Enamel (Mycerium Dental, France) [13]. The ENA HEAT composite warmer (Mycerium Dental, France) was used for optimal handling and adaptation per the manufacturer’s instructions. Before final curing, Air Block (Shiny G) was applied to reduce the oxygen-inhibited layer and ensure full polymerization. After polishing with the Cosshining-Y + G Polishing Paste Shiny Mini Kit (Mycerium Dental, France), the restorations were rescanned with the Medit i700 to assess final anatomy and consistency with the initial situation. The composition of the restorative material is in Table 1.

### 2.3. Orthodontic Aligners Manufacturing

Custom-made orthodontic aligners were made for each molar with a vacuum thermoforming machine and 0.75 mm thermoplastic sheets/foil (Scheu-Dental’s CA Pro, SCHEU-DENTAL GmbH, Iserlohn, Germany). The aligners were trimmed and fitted for optimal adaptation and retention. To simulate 6 months of treatment, each aligner was replaced every two weeks, for a total of 12 aligners per tooth. Each group had 17 molars, yielding 204 aligners per group, reflecting a two-week wear cycle as in clinical practice.

### 2.4. Masticatory Force Simulation

A dual-axis chewing simulator (CS 4.2, SD Mechatronik, Feldkirchen-Westerham, Germany) was used to replicate masticatory forces under ex vivo conditions. Before simulation, the roots of the restored teeth were wax-sealed apically, covered with type 3 polyvinyl siloxane (PVS), and embedded in self-cured acrylic resin. This followed a previously published model [14]. While undergoing mechanical loading and surface treatments, the restored teeth were immersed in artificial saliva (Sigma-Aldrich, a subsidiary of Merck KGaA, Darmstadt, Germany). The artificial saliva contained, per liter, 0.126 g NaCl, 0.964 g KCl, 0.189 g KSCN, 0.655 g KH_2_PO_4_, and 0.200 g urea [15].

The chewing simulator was set to reproduce vertical loading and lateral sliding movements. This simulated the occlusal forces and shear stresses seen during natural and parafunctional mastication.

The teeth were randomly assigned to the following groups:

Group 1: Normal chewing simulation—cyclic loads of 1 kgF (10 N) at a frequency of 1.6 Hz for 125,000 cycles, simulating normal masticatory forces over half a year.

Group 2: Parafunctional forces simulation—cyclic loads of 7 kgF (70 N) at a frequency of 1.6 Hz for 125,000 cycles, with lateral travel of ±1 mm simulating the increased forces associated with bruxism over half a year.

This load magnitude was selected to represent light to moderate parafunctional forces, as commonly reported in the literature [14,16]. It provided a realistic yet conservative approximation of clinical bruxism conditions. The chosen parameters ensured repeatability of the experiment while preventing excessive structural damage to specimens. This approach maintained relevance to average intraoral mechanical stress. The lateral sliding movement was set to 1 mm, replicating the shear forces observed during chewing. Each cycle included vertical force application followed by a lateral sliding motion. This ensured realistic replication of functional and parafunctional forces. The loading protocol has been previously validated and applied in other studies designed to simulate bruxism-related forces [14,16].

### 2.5. Surface Wear/Restoration Integrity Evaluation

#### 2.5.1. Restorative Material Assessment

**Figure 3:**
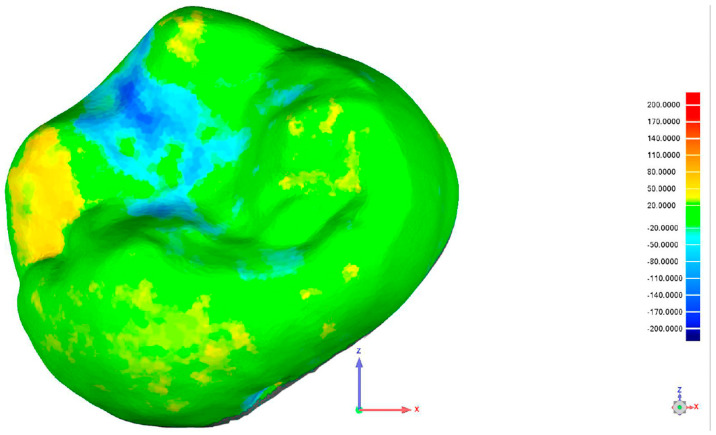
Superimposition of one of the specimens using Geometric Qualify Software, version Qualify 2013.

After the chewing simulation, a second intraoral scan was performed using the Medit i700. To evaluate surface wear and restoration integrity, the pre- and post-simulation digital models were superimposed with Geomagic Qualify software, version Qualify 2013 (Geomagic, Inc., Research Triangle Park, Morrisville, NC, USA). This enabled precise measurement of volumetric loss, as shown in Figure 3.

The figure below shows a color-coded surface deviation map. This map was created by overlaying the baseline scan (before force application) with the scan recorded after loading. Color variations indicate the magnitude of material loss or displacement: green areas show minimal or no change, yellow to red areas denote positive deviations (material loss), and blue areas represent negative deviations (surface depression or wear). This analysis allows quantitative assessment of substance loss caused by the applied forces.

#### 2.5.2. Aligner Integrity Analysis

**Figure 4:**
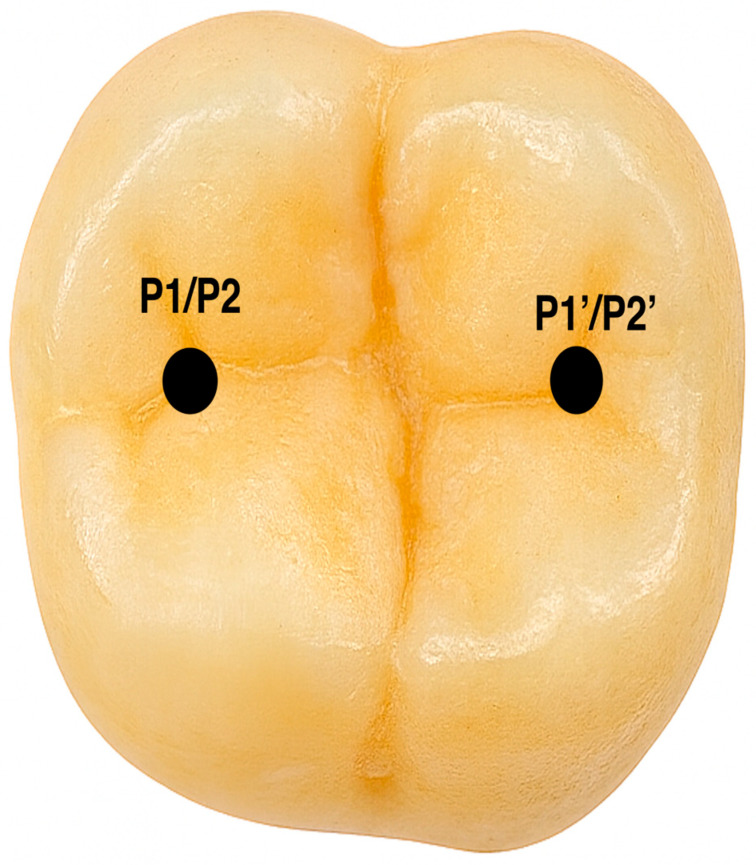
The locations where forces were applied and the thickness of the aligner were measured.

Aligner integrity was assessed with a digital caliper (Unimax—serial no. 8219), measuring thickness at two predefined sites before and after load application. Measurement points were directly on the aligner at the sites of load exposure. P1′ marked the area subjected to simulated parafunctional forces, while P1 was the opposing site not exposed to force, serving as a control. P2′ indicated the region subjected to physiological masticatory forces, and P2 its corresponding control site. Thickness changes at these locations indicated the mechanical impact of different loading conditions on the aligner material (see Figure 4).

### 2.6. Statistical Analysis

The collected experimental data were analyzed using R, version 4.3 [17], under the Jamovi project version 2.5.3 interface [18]. Differences between the two groups were assessed using paired samples *t*-tests. These results were confirmed using Wilcoxon tests for paired samples and robust paired samples *t*-tests when data normality could not be confirmed by Q-Q plots and normality tests (Shapiro–Wilk and Anderson–Darling). The significance level for all tests was set at α = 0.05.

To strengthen the reliability of the primary findings, robust paired samples t-tests were conducted to minimize the influence of potential outliers and non-normal distributions. In this study, Root Mean Square (RMS) error was used as a quantitative indicator to assess the impact of simulated parafunctional forces on Class II restored teeth. RMS error measures the average magnitude of deviation from a reference, reflecting stress distribution or displacement under repetitive loading. In our specific context, RMS error was calculated by superimposing the initial and final 3D scans of the restored molars before and after the application of simulated parafunctional forces. The resulting values reflected volumetric changes at the level of the restorative material, allowing us to objectively quantify the material loss or degradation associated with functional overload.

## 3. Results

Table 2 shows the RMS errors measured under two conditions: normal function (RMS error F) and simulated extra forces (RMS error PF). The average and middle RMS errors, with their 95% confidence ranges, are shown. In normal conditions, the average RMS error was much lower than when extra forces were added (*p* < 0.05). For normal function, the average RMS error was 21.5 units, and the middle value was 21.9, with a spread of 1.82 and a margin of 0.469, which shows the samples were similar. When extra forces were used, the average became 33.5, the middle value was 32.8, the spread was 4.90, and the margin was 1.266, showing more varied results.

Mean and median RMS errors were higher under simulated parafunctional conditions, indicating increased positional inaccuracies due to these forces (Table 2).

The statistical analyses reinforce this observation. The paired samples Student’s t-test indicates a highly significant mean difference of −11.9 (*p* < 0.001, 95% CI: −14.9 to −8.92). These results were further confirmed by the Wilcoxon paired samples test. This test yielded a mean difference of −11.3 (*p* < 0.001, 95% CI: −14.6 to −8.63).

**Figure 5:**
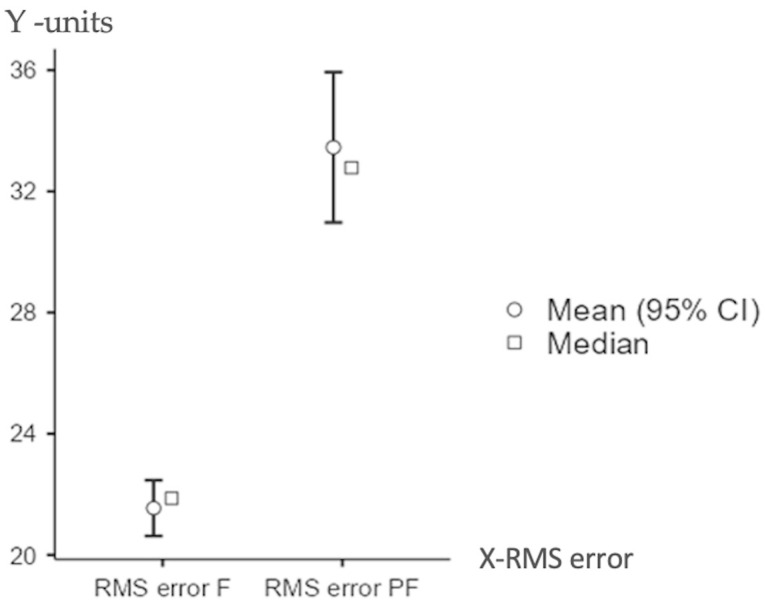
Distribution of RMS error under functional vs. parafunctional forces.

Table 3 shows the distribution of RMS error values under both functional and parafunctional loading conditions. The boxplot illustrates a significantly higher median for the parafunctional group (PF) compared to the functional group (F). The interquartile range is also wider for PF (*p* < 0.05) (Figure 5).

Descriptive statistics provided additional insights into the distribution and variability of aligner thickness changes under different loading conditions. At the P1′ site (parafunctional forces), the mean thickness reduction was 0.0304 mm, with a median of 0.0305 mm, a standard deviation (SD) of 0.00719, and a standard error (SE) of 0.000504.

Under P2′ (functional forces), the mean reduction was 0.0122 mm, with a median of 0.0120 mm, an SD of 0.00350, and an SE of 0.000245, reflecting a more limited but consistent degree of material wear.

For the P1 (control group for parafunctional forces), the mean thickness change was 0.00094 mm, with a median of 0.00100 mm, SD of 0.000671, and SE of 0.0000470. Similarly, for the P2 (control group for functional forces), with a mean of 0.00085 mm, median of 0.00100 mm, SD of 0.000672, and SE of 0.0000470.

At site P1′ (parafunctional loading), the mean thickness reduction was 0.0304 mm (SD = 0.00719), which was significantly higher than the control value of 0.00094 mm (SD = 0.00067). The difference was highly significant (*t* = 58.3, *p* < 0.001), with a very large effect size (Cohen’s *d* = 4.08). The Wilcoxon signed-rank test confirmed this result (*W* = 20910, *p* < 0.001), with a rank biserial correlation of 1.00, indicating a consistent direction of the effect across all samples.

**Figure 6:**
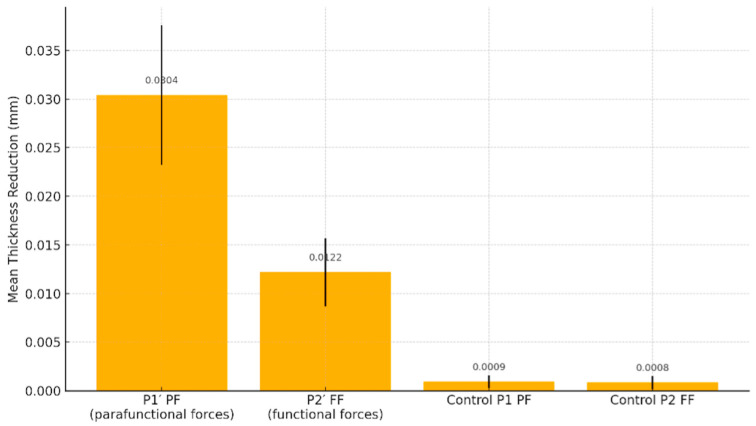
Aligner thickness reduction under functional vs. parafunctional loading.

At site P2′ (functional loading), the mean reduction was 0.0122 mm (SD = 0.00350), also significantly greater than the control value of 0.00085 mm (SD = 0.00067). This difference was statistically significant (*t* = 45.6, *p* < 0.001), with a large effect size (Cohen’s *d* = 3.19). The Wilcoxon test again supported the result (*W* = 20910, *p* < 0.001), with a rank biserial correlation of 1.00 (Figure 6).

A direct comparison between parafunctional and functional loading sites (P1′ vs. P2′) revealed a statistically significant difference in material loss. The mean difference was 0.0181 mm (SD = 0.00056), with *t* = 32.7 and *p* < 0.001. This contrast yielded a strong effect size (Cohen’s *d* = 2.29), demonstrating that parafunctional forces produced significantly more wear in the aligner material than physiological forces.

Normality tests (Shapiro–Wilk, Kolmogorov–Smirnov, and Anderson–Darling) revealed slight deviations from normal distribution in some conditions; however, consistent results across both parametric and non-parametric tests confirm the robustness and statistical reliability of the findings.

The robust analysis yielded statistically significant results for both experimental groups. In the P1′ PF group, the adjusted mean difference relative to control was −0.0294 mm (*p* < 0.001, 95% CI: −0.0306 to −0.0281), while in the P2′ FF group, the robust mean difference was −0.0115 mm (*p* < 0.001, 95% CI: −0.0121 to −0.0109).

Furthermore, tests of distributional assumptions revealed mild deviations from normality in some comparisons. The Shapiro–Wilk test showed this most clearly (*p* = 0.003 for P1′ PF vs. control, and *p* < 0.001 for P2′ FF vs. control). Despite these deviations, complementary evidence from the Kolmogorov–Smirnov and Anderson–Darling tests supports the data’s reliability. The agreement between parametric and non-parametric analyses further demonstrates the robustness and internal consistency of the dataset. These outcomes validate the statistical soundness of the comparisons, especially with clinical biomaterials subject to variable load responses.

**Figure 7:**
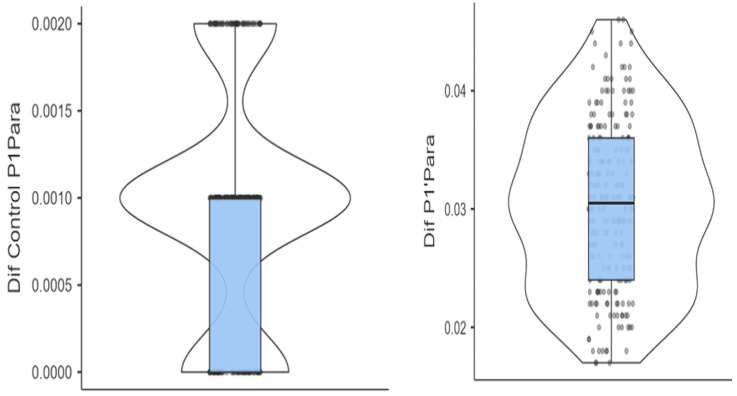
Box and violin plots contrasting the differences in aligner thickness between the test and control groups.

These results are shown in Figure 7. The figure illustrates the mean thickness differences between experimental and control groups and provides their 95% confidence intervals. This visual confirms both the statistical strength and clinical relevance of the observed effects.

Median differences in thickness are represented by the central horizontal lines. Boxes represent the interquartile ranges (IQRs). Upper and lower whiskers mark the minimum and maximum differences in aligner thickness, respectively.

## 4. Discussion

Current ex vivo evidence suggests clear aligners have adequate durability under normal intraoral conditions, with only minor reductions in thickness and mechanical force over time [19]. Simulated bruxism accelerates wear and reduces performance, but aligners generally withstand substantial loading before failure [20]. Several studies have investigated aligner stability under repeated functional and parafunctional stress. Albertini et al. [21] showed significant stress relaxation in thermoplastic aligners over 14 days, indicating gradual force loss from viscoelastic behavior. Xiang et al. [22] found that aligners immersed in artificial saliva deliver less force over time, with modified PET-G materials outperforming conventional ones. Dalaie et al. [23] reported that a higher glass transition temperature (Tg) improves post-thermoforming and aging stability, highlighting Tg’s relevance for durability. Evidence suggests that temperature, pH, and enzymatic activity may accelerate surface wear and microstructural degradation in aligner polymers [24,25]. Collectively, these findings reinforce the need to assess degradation and performance under both functional and parafunctional loading. Advances in design, such as multi-layer composites and improved formulations, enhance stress retention and wear resistance. Such developments are crucial for managing patients with parafunctional masticatory status, where protocols may need adaptation to maintain effectiveness while minimizing excessive occlusal forces.

The present study aimed to evaluate the wear and fracture patterns of the restorations and the potential effects of aligner wear on restoration integrity under clinically relevant forces.

Based on the results of the present study, both null hypotheses were rejected. The significantly greater differences observed under parafunctional loading indicate that these forces exert a more detrimental effect than functional forces, both on the restored teeth and on the aligners themselves.

### 4.1. Impact of Parafunctional Forces on the Integrity of Composite Restorations

Resin composites are primarily composed of resin matrices reinforced with inorganic fillers. These materials are designed to withstand functional occlusal forces during normal mastication [26,27]. However, the filler–resin matrix interface is particularly vulnerable to degradation under parafunctional loading—such as the cyclic, high-magnitude forces seen in bruxism. Such forces promote microfractures, matrix fatigue, and filler particle loss. Over time, these effects result in structural compromise and surface deterioration [28,29]. 

Our findings demonstrated statistically significant differences in restoration integrity between teeth exposed to simulated bruxism and those under normal occlusal loading. This indicates that parafunctional forces accelerate material fatigue and the failure of restorations. This finding is consistent with previously published results [30,31]. Those studies showed that bruxism increases marginal gap formation and susceptibility to secondary caries due to repeated sub-catastrophic loading.

Excessive occlusal forces have been linked to increased surface roughness, bond degradation, and marginal deterioration. This is especially true in posterior restorations subjected to chronic stress [32,33]. Cayo-Rojas et al. [34] also reported a higher occurrence of wear and microleakage in composite restorations among bruxism patients. This supports the idea that parafunctional habits create a high-risk environment for restorative failure [31].

Studies show that nanohybrid and conventional resin composites subjected to bruxism-like loading exhibit a significant rise in interfacial defects and mechanical breakdown. Hamburger et al. [35] reported that simulated bruxism conditions result in increased fracture lines along the tooth–restoration interface, even in high-performance composites. Similarly, Zhang et al. emphasized that even with advanced formulations, current resin composites remain susceptible to fatigue under prolonged occlusal overloads. These findings suggest the need for cautious material selection and mechanical reinforcement in high-risk patients [36,37].

The mechanical properties of restorative composites, particularly filler content and polymer cross-linking, directly influence their clinical longevity under load [33]. High filler volume enhances resistance to wear and increases the elastic modulus. Still, it may not entirely prevent degradation when restorations are subjected to chronic high-magnitude loads [16,38,39].

Parafunctional activities have also been linked to higher rates of Class V restoration fractures and adhesive failures [40]. Our study found that under simulated bruxism conditions, Class II composite restorations showed increased surface degradation and mechanical instability. These findings align with prior evidence. Previous studies showed that parafunctional loading leads to significantly more restoration defects than functional occlusion alone [41].

These descriptive values are consistent with the higher mechanical stress induced by parafunctional forces, as shown by the increased variability. The observed shift in both central tendency and spread reinforces the clinically relevant impact of parafunction on restoration performance. Parafunction should be considered a key risk factor when planning posterior composite restorations.

Therefore, the first null hypothesis was rejected. It is assumed that simulated parafunctional forces have a comparable influence to functional forces on the integrity of Class II restored teeth covered by orthodontic aligners. Parafunctional loading produced significantly greater surface degradation, marginal deterioration, and loss of restoration stability compared to functional forces.

### 4.2. Material Performance and Aligner Selection

Single-layer aligners are made from uniform thermoplastic materials like PET-G or TPU. While effective, they tend to lose force over time due to viscoelastic relaxation and are more prone to surface wear and deformation. Multi-layer aligners feature a flexible core between rigid outer layers, offering better force retention, durability, and resistance to fatigue [42,43]. Their composite structure also performs better in moist or acidic oral environments. Clinically, single-layer aligners suit patients with lower bite forces, while multi-layer designs are more stable and reliable for those with higher masticatory loads or longer wear durations [44].

CA^®^ Pro(Scheu-Dental’s CA Pro, SCHEU-DENTAL GmbH, Iserlohn, Germany), utilizes a hybrid-layer design that integrates elastomeric and rigid components. This approach allows better energy absorption and stress distribution during repeated occlusal loading. In a recent article, Šimunović L et al. [45] tested different brands of aligners in controlled laboratory conditions. The results showed that multi-layer aligners, like the one used in this study, exhibited lower permanent deformation, more stable force, and improved crack resistance over time. These were observed when compared to conventional aligners such as Invisalign SmartTrack^®^ (Align Technology, Inc., San Jose, CA, USA) and Zendura FLX^®^ (Bay Materials LLC, Fremont, CA, USA) [45,46]. These benefits are particularly relevant for patients with high occlusal force profiles or known parafunctional habits. In these cases, load attenuation and shape memory retention are key to preserving therapeutic efficacy [4].

Furthermore, extending the explanation of these material advantages, the layered structure of the aligners supports delayed plastic deformation by dissipating energy across interfaces, reducing localized stress concentration, which is a common failure mode in single-layer thermoplastics [21,47].

Given these characteristics, clinicians should prioritize the selection of advanced multi-layer aligner materials for patients with parafunctional activity. Clinical protocols should explicitly weigh material resilience, case complexity, wear patterns, and patient compliance to maximize aligner longevity and treatment outcomes [48,49]. This proactive approach can help mitigate the long-term risks associated with parafunctional stress.

Bruxism results in excessive occlusal loads ranging from 70 to 500 N or higher. These loads can deform, crack, or abrade aligners, leading to reduced fit and treatment effectiveness [50]. Microstructural analyses have confirmed surface degradation, internal stress buildup, and a measurable loss of mechanical properties after repeated cyclic loads. These effects are especially prominent in the posterior occlusal zones where force intensity is greatest. Recent investigations by Siotou et al. (2025) and Li et al. (2022) [4,51] have also demonstrated that cyclic loading leads to significant surface degradation and loss of mechanical strength in orthodontic aligner materials, particularly in areas exposed to high occlusal stress [51]. Our findings are consistent with these reports. They confirm that repetitive functional and parafunctional forces can accelerate material fatigue and deformation. In restorative and orthodontic practice, these results highlight the need to select aligner materials with enhanced mechanical resilience and to monitor patients with parafunctional habits more closely.

Although aligners may temporarily buffer occlusal forces and reduce immediate load transmission to dental restorations [52], this protective effect is limited under bruxism conditions. The aligners themselves become subject to material fatigue and plastic deformation within days of exposure to excessive cyclic stress [53]. These descriptive values match the higher mechanical stress caused by parafunctional forces. This is evident in the increased variability observed. The shift in both central tendency and spread highlights the clinically relevant impact of parafunction on aligner performance.

The durability of clear aligners depends on several factors, including thickness, polymer composition, and the thermoforming process used during manufacturing. Thinner aligners deform more easily and break down mechanically under stress [54,55]. The thermoforming process can cause polymer chain relaxation and uneven stress distribution. This ultimately decreases strength, flexibility, and force delivery capacity [56].

Additional mechanical degradation occurs from routine clinical use. Frequent insertion and removal during wear cycles introduce surface microcracks, delamination zones, and edge deformation. These defects worsen material fatigue under functional or parafunctional loads [57,58]. Clear aligners exposed to bruxism-like loading quickly lose stiffness and force accuracy. Substantial structural changes appear even after short periods of simulated use [59,60].

The second null hypothesis of our study assumed that parafunctional and functional forces have a comparable influence on the integrity of orthodontic aligners. This hypothesis was rejected, as aligners exposed to simulated bruxism showed higher levels of thickness reduction, deformation, and mechanical fatigue than those subjected to functional loading. These findings underscore the need for careful material selection, optimal aligner thickness, and possibly the adjunctive use of occlusal splints for bruxism patients undergoing aligner therapy. These factors are critical for ensuring the long-term clinical success of both composite restorations and orthodontic aligner therapy in bruxism patients. They should guide clinical decision-making in this high-risk population.

### 4.3. Study Limits and Future Research

This study was conducted under ex vivo conditions, which do not fully replicate the complexity of the oral environment and the variability of real-life parafunctional forces. Additionally, the selection of materials and loading parameters was based on standardized models, which may limit the direct clinical applicability of the findings. Only one type of resin composite and one brand of multi-layer aligner were tested, representing an additional methodological limitation. Different composites and aligner materials may respond differently under similar loading conditions, which should be explored in future studies.

Future research should focus on developing advanced materials with enhanced wear resistance and fatigue properties, specifically tailored for use in bruxism patients. Additionally, future studies are needed to investigate other critical characteristics, such as surface roughness and flexural strength [61], which would broaden the understanding of material performance under functional and parafunctional conditions. Moreover, as highlighted by España-Pamplona et al., the variation in the oral microbiota within aligners represents a fundamental aspect of treatment [62]. Therefore, future research should also explore how functional and parafunctional forces may influence microbiota composition and dynamics during aligner therapy. Emphasizing these aspects will help define a clearer roadmap for future investigations, underscoring the novelty of the present work while situating it within a broader research framework. The combination of in vitro testing and long-term clinical studies will provide deeper insights into optimizing restorative and orthodontic outcomes in this patient population.

## 5. Conclusions

Within the limitations of the study, it was concluded that parafunctional forces significantly accelerate surface wear in resin composite restorations—particularly in Class II posterior fillings—when patients are undergoing orthodontic treatment with clear aligners. This finding highlights the need for enhanced restorative strategies in patients with bruxism. Additionally, simulated bruxism forces were found to compromise the integrity of aligners, leading to observable surface alterations and material fatigue after short exposure durations, especially in single-layer thermoplastic aligners. However, these in vitro findings should be clinically confirmed through long-term studies to validate their relevance under real intraoral conditions.

## Figures and Tables

**Table 1:** The composition of the restorative materials used in the study.

Material	Manufacturer	Monomer Matrix	Filler Content	Lot Number
ENA HRi BioFunction Dentin	Micerium S.p.A. (Avegno, Italy)	Urethane dimethacrylate (UDMA), tricyclodecane dimethanol dimethacrylate (TCDDM)	74% by weight (60% by volume); 0.005–0.05 µm silicon dioxide and 0.2–3.0 µm glass fillers	2019008149
ENA HRi BioFunction Enamel	Micerium S.p.A., (Avegno, Italy)	Urethane dimethacrylate (UDMA), tricyclodecane dimethanol dimethacrylate (TCDDM); BPA-free, Bis-GMA-free	74% by weight (60% by volume); nanoparticle aggregates, fluoride-releasing, high radiopacity (~250% Al)	2021002871

**Table 2:** Descriptive statistics of RMS error.

Measure	Mean	Median	SD	SE
RMS Error F	21.5	21.9	1.82	0.469
RMS Error PF	33.5	32.8	4.90	1.266

**Table 3:** Confidence intervals (CIs) of the mean differences for RMS error F and RMS error PF.

Comparison	Test	Statistic	df	*p*	Mean Difference	SE Difference	95% CI Lower	95% CI Upper
RMS error F vs. RMS error PF	Student’s t	−8.54	14.0	<0.001	−11.9	1.39	−14.9	−8.92
RMS error F vs. RMS error PF	Wilcoxon W	0.00		<0.001	−11.3	1.39	−14.6	−8.63

Note. H_a_ μ Measure 1 − Measure 2 ≠ 0.

## Data Availability

The data supporting this study’s findings are available on request from the corresponding author.
